# Classification of Hippocampal Ripples: Convolutional Neural Network Learns Episode-Specific Changes

**DOI:** 10.3390/brainsci14020177

**Published:** 2024-02-14

**Authors:** Yuta Ishihara, Ken’ichi Fujimoto, Hiroshi Murai, Junko Ishikawa, Dai Mitsushima

**Affiliations:** 1 Graduate School of Science for Creative Emergence, Kagawa University, Kagawa 761-0396, Japan; s23g403@kagawa-u.ac.jp; 2 Faculty of Engineering and Design, Kagawa University, Kagawa 761-0396, Japan; 3 Faculty of Global and Science Studies, Yamaguchi University, Yamaguchi 753-8541, Japan; muraip@yamaguchi-u.ac.jp; 4 Graduate School of Medicine, Yamaguchi University, Yamaguchi 755-8505, Japan; junko-lc@yamaguchi-u.ac.jp

**Keywords:** ripple firings, episodic experience, classification, convolutional neural network, gradient-weighted class activation mapping (Grad-CAM), t-distributed stochastic neighbor embedding (t-SNE)

## Abstract

The hippocampus is known to play an important role in memory by processing spatiotemporal information of episodic experiences. By recording synchronized multiple-unit firing events (ripple firings with 300 Hz–10 kHz) of hippocampal CA1 neurons in freely moving rats, we previously found an episode-dependent diversity in the waveform of ripple firings. In the present study, we hypothesized that changes in the diversity would depend on the type of episode experienced. If this hypothesis holds, we can identify the ripple waveforms associated with each episode. Thus, we first attempted to classify the ripple firings measured from rats into five categories: those experiencing any of the four episodes and those before experiencing any of the four episodes. In this paper, we construct a convolutional neural network (CNN) to classify the current stocks of ripple firings into these five categories and demonstrate that the CNN can successfully classify the ripple firings. We subsequently indicate partial ripple waveforms that the CNN focuses on for classification by applying gradient-weighted class activation mapping (Grad-CAM) to the CNN. The method of t-distributed stochastic neighbor embedding (t-SNE) maps ripple waveforms into a two-dimensional feature space. Analyzing the distribution of partial waveforms extracted by Grad-CAM in a t-SNE feature space suggests that the partial waveforms may be representative of each category.

## 1. Introduction

The hippocampus plays a pivotal role in the formation of new episodic memories across various mammalian species, including humans [1]. Hippocampal neurons appear to process a diverse range of information such as spatial location [2,3], temporal information [4], and emotional state [5] within specific episodes [6]. Although the critical mechanism of how a piece of a specific memory is maintained or what connects the memory fragments is still largely unknown, CA1 neurons in the dorsal hippocampus are known to be required for contextual memory [7], spatial learning [8,9], and object recognition [9]. Notably, the dorsal CA1 region houses a considerable number of junction-place cells that encode the location of other animals within the same cage [10].

While the ventral hippocampus [11] and CA2 neurons [12] are recognized for their essential role in social memory, this study specifically focuses on the dorsal CA1 region. This region responds to diverse experiences and diversifies synapses to facilitate learning [13,14], allowing for the analysis of changes specific to episodic experiences.

Hippocampal ripple oscillations (140–250 Hz) are oscillatory patterns observed in electroencephalograms that are essential for memory and action planning [15,16]. Since brief high-frequency synchronous firings are known to occur in conjunction with ripple oscillations [17], we recorded a band of 300 Hz–10 kHz to detect the firing activity of adjacent multiple neurons behind a single ripple in dorsal CA1 [14]. In a single event of ripple firings, the multiple neurons fired synchronously for a brief period of approximately 20 to 150 msec, mostly co-occurring with the sharp-wave ripple. In dorsal CA1, suppression of sharp-wave ripples impairs learning and memory [8,18], and learning prolongs the duration [19]. A firing sequence during a sharp-wave ripple replays the sequence of locations during spatial learning [20], and spikes during a sharp-wave ripple increase with learning [19]. These observations suggest that ripples contain learning information, leading us to hypothesize that ripple firings may represent part of prior experiences.

To test this hypothesis, we previously conducted a comprehensive analysis of thousands of ripple firings occurring before and after episodic experiences. We found episode-dependent changes and episode-specific differences in the diversity expressed by the four features (amplitude, duration, number of spikes, and arc length) [14]. If our hypothesis is confirmed, we may be able to identify specific waveforms or significant information related to experienced episodes from ripple firings using artificial neural networks that can learn representations from the electroencephalogram [21,22]. 

Convolutional neural networks (CNNs) are widely used in image recognition, pattern recognition, and natural language processing [23]; CNNs use the principles of linear algebra, particularly matrix multiplication, to identify patterns in images, and they have the potential to classify ripple firings associated with each episode. As a first step toward confirming this hypothesis, the aim of this paper is to classify the ripple firings measured from rats that experienced four types of episodes into five categories: experiencing one of the four episodes or before an episodic experience. We construct a CNN to classify the current stocks of ripple firings into the five categories and demonstrate that the CNN classifies them well.

Subsequently, by applying gradient-weighted class activation mapping (Grad-CAM) [24], which is an explainable artificial intelligence (XAI) technique, to the CNN, we analyze partial waveforms that the CNN focuses on for classification and extract the partial waveforms. The t-distributed stochastic neighbor embedding (t-SNE) [25] can map ripple waveforms onto points in a two-dimensional (2D) feature space. We compare the distribution of ripple waveforms input to the CNN and partial waveforms extracted by Grad-CAM in feature spaces created by t-SNE. The analyzed results using Grad-CAM and t-SNE suggest that the extracted partial waveforms may be representative of each category.

## 2. Materials and Methods

### 2.1. Animals and Surgery

Male Sprague-Dawley rats (CLEA Japan Inc., Tokyo, Japan) aged 15–25 weeks old were used for the recording of multiple-unit firing activity (MUA) of hippocampal CA1 neurons. Each rat was kept in a cage where the temperature was controlled to 24 ± 1 °C and the light was on for 12 h (from 8 a.m. to 8 p.m.). All rats were housed individually and had no contact with other rats for several weeks prior to the recording of MUAs. These rats were fed ad libitum at least two weeks before surgery (MF, Oriental Yeast Co. Ltd., Tokyo, Japan). For the episodic stimuli, 8- to 15-week-old male or female rats were separately prepared without electrode implantation; two to three same-sex rats were housed and kept separately from the rats for the recording. 

**Figure 1:**
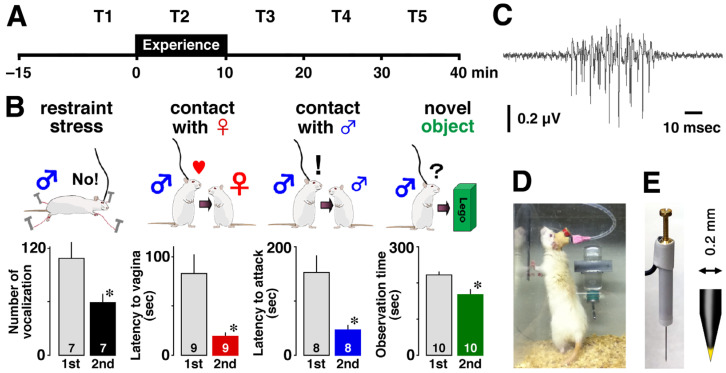
**Confirmation of experimental episodic memory and MUA recording in CA1.** (**A**) Recording schedule and 10 min episode. (**B**) Cartoons illustrating the four types of episodic experiences. After the episodic experiences (1st), memory acquisition was assessed for each experience the following day (2nd). Data are the mean ± SEM (standard error of the mean). The number of rats for the behavioral study is shown at the bottom of each bar. * *p* < 0.05 vs. 1st. (**C**) Example of a ripple firing. Multiple spikes form a single event. (**D**) Picture of a recorded animal. (**E**) A movable recording electrode with an enlarged tip.

Prior to the experiment, animals were anesthetized with sodium pentobarbital (50 mg/kg, intraperitoneal) and placed in a stereotaxic apparatus. Vertically movable tungsten recording electrodes with a resistance of 50 to 80 kΩ (Figure 1E: KS-216, Unique Medical Co., Ltd., Tokyo, Japan) were chronically implanted just above the CA1 area of the dorsal hippocampus (posterior, 3.0–3.6 mm; lateral, 1.4–2.6 mm; ventral, 2.0–2.2 mm) and fixed with dental cement. Rats were kept in their home cages for at least one week to recover from the implantation surgery (Figure 1D).

### 2.2. Measurement of MUAs

On the day of the experiment, the electrode was carefully inserted into the CA1 pyramidal cell layer without anesthesia, and the recording was started in their familiar home cage. To monitor the encoding process of the experience, we recorded multiple-unit firing activity of CA1 before (15 min), during (10 min), and after (30 min) each episode (Figure 1A) using an electrode that could record neural activities from many neighboring neurons (Figure 1E). To mimic the events in humans that lead to episodic memory, the rats were exposed for 10 min to one of four different episodes: restraint stress, social interaction with a female or a male, and observation of a novel object (Figure 1B). For each rat, we recorded MUAs for 55 min before and after a given event, and the time slots were labeled as T1, T2, T3, T4, and T5 (Figure 1A). All experiments were performed during the light period. The number of rats in restraint, contact with a female, contact with a male, and contact with a novel object was 7, 8, 8, and 7, respectively.

We extracted thousands of ripple firings (Figure 1C) from all recorded MUAs according to reported criteria [14]. Neural signals were passed through a head amplifier and then through a shielded cable into the main amplifier (MEG-2100 or MEG-6116; Nihon Kohden, Tokyo, Japan), and the reference level for MUAs was the cortical potential. Signals were band-pass filtered at 150–10 kHz and digitized using a CED 1401 interface controlled by Spike2 software (Cambridge Electronics Design, Cambridge, UK). All signal data were sampled at 25 kHz. Recorded signals (150 Hz–10 kHz) were filtered at 150–300 Hz and 300 Hz–10 kHz, with 150–300 Hz used for sharp-wave ripple detection and 300 Hz–10 kHz signals used for firing analysis.

Using the 150–300 Hz signal, we detected sharp-wave ripples by calculating the root mean square and setting the threshold for event detection at +6 SD above the mean of the baseline. Because most of the ripple firings co-occurred with the sharp-wave ripples, we used the 300 Hz–10 kHz signal to analyze the firing behind the sharp-wave ripple (i.e., ripple firings) in the present study. A ripple firing, which counted the number of occurrences, was defined as one with a short duration (55.6 ± 0.3 msec, n=4569) and a signal-to-noise ratio of at least six to one (Figure 1C).

Isolation of single units was initially performed using the template-matching function of the Spike2 software. As previously reported [26], all spikes used in the subsequent analysis were clearly identified, with a signal-to-noise ratio of at least three to one. After the initial spike separation, we applied principal component analysis to the detected waveforms. However, in this experiment, the sorting was not always reliable, especially in ripple firings, because one electrode recorded many units. Therefore, we analyzed all recording data as multiple unit firing activity. The waveform characteristics of the spikes showed that the recorded spikes were mostly formed by pyramidal cells and some interneurons, confirming that the recording site was in the pyramidal cell layer.

Not only the shape of spikes, but we also further verified the location of recording site histologically. At the end of the experiments, animals were deeply anesthetized with sodium pentobarbital (400 mg/kg, i.p.) and immediately perfused transcardially with a solution of 0.1 M phosphate buffer containing 4% paraformaldehyde. The brain was removed and then post-fixed with the same paraformaldehyde solution and immersed in 10–30% sucrose solution. Coronal sections (40 μm thick) were stained with hematoxylin and eosin. The locations of cannulas, recording electrode tips, and tracks in the brain were identified using a stereotaxic atlas [27].

### 2.3. Confirmation of Experimental Episodic Memory

To assess acquired memory, the rats were re-exposed to the same episode for 5 min and their behavior was monitored. Rats that experienced restraint stress showed fewer audible vocalizations during the second exposure ($t_{6}=3.476,p=0.0129$). Similarly, rats exposed to a female, male, or novel object consistently reduced latency to vaginal inspection ($t_{8}=3.492,p=0.0082$) or attack ($t_{7}=4.192,p=0.0041$) and object observation time ($t_{9}=2.901,p=0.0176$) during the second encounter, suggesting memory acquisition (Figure 1B).

### 2.4. Preprocessing of Ripple Firings

We measured a lot of MUAs and visually extracted thousands of ripple firings from the MUAs. All the ripple firings can be annotated by types of experienced episodes or before episode experience. However, ripple firings with a rich diversity [14] may reduce the effectiveness to find representative waveforms in each episode or before episodic experience. As preprocessing for analyzing ripple firings, we proposed a method to automatically extract only ripple firings with similar waveforms based on standardization, logarithmic transformation, and a cross-correlation function that is a measure of similarity between two waveforms. Before the preprocessing, we removed too short ripple firings, less than 15 msec, because they may reduce the accuracy of classification of ripple firings. The details of the proposed method are as follows.

The cross-correlation function is often used in neuroscience [28], and it can measure the similarity even for two ripple firings of different time lengths. Moreover, it can calculate the similarity well even if the timing of the appearance of episode-dependent local waveforms differs. Now, let $x_{i}(t)$ and $x_{j}(t)$ be the amplitude of the ith and jth ripple firings at time t. The similarity between $x_{i}$ and $x_{j}$ based on cross-correlation function can be computed as follows:

(1) $$C_{x_{i}x_{j}}=\underset{\tau }{\max}\sum_{t}x_{i}\left(t\right)x_{j}(t-\tau ),\;\;\;\;\;\;\;\;i\neq j$$

where τ represents the time shift. In addition, since the lengths of ripple firings are generally different, we normalize the $C_{x_{i}x_{j}}$ value by dividing by $C_{x_{i}x_{i}}$, which is called auto-correlation function, so that the normalized value takes roughly between 0.0 and 1.0. When it takes a high value near 1.0 or more, the two ripple firings are similar. Although this normalization is not common, it can pick up partially similar ripple firings even if the length of waveforms is quite different. This is because our normalization can prevent the degradation of cross-correlation function values with significantly different lengths.

Despite the same episodic experience, the amplitude of measured MUAs may vary depending on individual rats. Since such a difference in amplitude can reduce the accuracy of the classification of ripple firings, we perform preprocessing to minimize the difference. Let $x_{i}$ be the amplitude of the ith ripple firings. Standardization (z-score normalization) is defined as follows:

(2) $$y_{i}\left(t\right)=\frac{x_{i}\left(t\right)-m_{i}}{\sigma_{i}},$$

where t denotes the discrete time within the extracted ripple firings and $m_{i}$ and $\sigma_{i}$ represent the mean and standard deviation of $x_{i}$, respectively.

**Figure 2:**
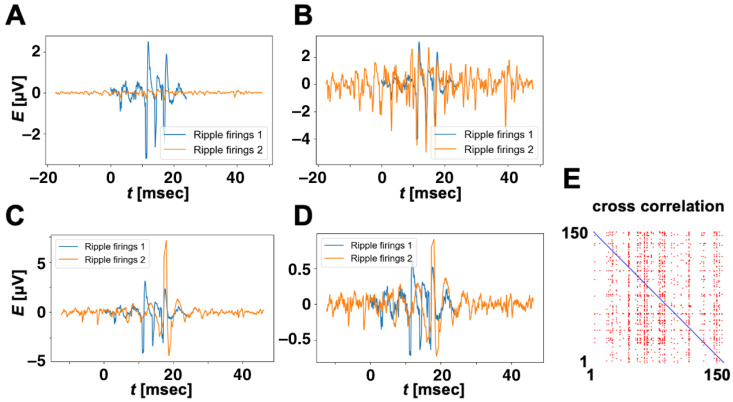
**Effect of standardization and logarithmic transformation.** (**A**) Ripple firings without standardization. Blue and orange waveforms correspond to ripple firings of the second and tenth rats. (**B**) Waveforms after standardization for ripple firings in (**A**). (**C**) Standardized ripple firings without logarithmic transformation. Blue and orange waveforms correspond to ripple firings of the first and sixth rats. (**D**) Waveforms after logarithmic transformation for standardized ripple firings in (**C**). (**E**) Visualized values of cross-correlation function for 150 ripple firings with colors. The column and row axes correspond to $z_{i}$ and $z_{j}$. The blue pixels in the diagonal and red pixels mean the values of the cross-correlation function are 1.0 and more than 0.6, respectively.

Figure 2A,B show the results of standardization for two ripple firings with a big difference in amplitude. In Figure 2A, the blue and orange waveforms are ripple firings, $x_{1}$ and $x_{2}$, recorded from the second and tenth rats, respectively, i.e., without standardization. The two waveforms are not similar at first glance, and, besides, the normalized $C_{x_{1}x_{2}}$ value by the $C_{x_{1}x_{1}}$ value is quite small at 0.08. Standardization can reveal invisible similarity between the two waveforms as shown in Figure 2B. The normalized $C_{y_{1}y_{2}}$ value by the $C_{y_{1}y_{1}}$ value becomes 0.98.

When there are one or more large spikes in standardized ripple firings, the similarity analysis using the cross-correlation function can give a wrong decision. Figure 2C shows standardized ripple firings $y_{3}$ (blue) and $y_{4}$ (orange) for the first and sixth rats. In spite of that, the waveforms are not visually similar, and the normalized $C_{y_{3}y_{4}}$ takes a high value of 0.66. To avoid such a wrong decision in similarity analysis, we apply a logarithmic transformation to standardized ripple firings. It is defined as follows:

(3) $$z_{i}\left(t\right)=\left\{\begin{array}{cc} \log_{10}\left(y_{i}\left(t\right)+1\right) & if\;y_{i}\left(t\right)>0\\ 0 & if\;y_{i}\left(t\right)=0\\ -\log_{10}\left|y_{i}(t)-1\right| & if\;y_{i}\left(t\right)<0 \end{array}\right..$$

Figure 2D shows the waveforms after logarithmic transformation performed for standardized ripple firings in Figure 2C. The normalized $C_{y_{3}y_{4}}$ value is reduced to 0.48. Thus, logarithmic transformation not only makes a big spike small but also reduces the similarity value between two waveforms even when one or the other waveform contains one or more big spikes.

Figure 2E shows visualized values of the cross-correlation function with colors. The column and row axes correspond to $z_{i}$ and $z_{j}$, and the size of the image is 150×150 pixels. Although we have over 3000 ripple-firing stocks, we visualized the values of the cross-correlation function using only 150 stocks to make the image easier to view. The blue pixels in the diagonal and red pixels mean the values of the cross-correlation function are 1.0 and more than 0.6, respectively. Here, we set the threshold value to 0.6 and pick up only similar ripple firings such that the normalized $C_{z_{i}z_{j}}$ value is greater than the threshold value, i.e., only ripple firings with one or more red pixels in each column are picked out. The careful selection of ripple firings is useful for finding episode-dependent waveforms using a CNN and Grad-CAM, as detailed later.

### 2.5. Convolutional Neural Network

In general, a CNN has N layers, which are an input layer, some hidden layers, and an output layer. When a CNN is applied to classification problems in waveform datasets, the input layer has as many artificial neurons (mathematical models) as input data, and the neurons are arranged in a 1D grid. Neurons in a hidden layer are arranged in a 1D, 2D, or 3D grid, and the output layer has as many neurons as categories to be classified; the neurons are arranged in a 1D grid.

The hidden layers are generally composed of convolution layers, pooling layers, and fully connected layers. At the nth layer in the input and/or hidden layers, the ith neuron outputs an activated value with an activation function such as rectified linear unit (ReLU) defined as follows:

(4) $$z_{i}^{n}\left(y_{i}^{n}\right)=\left\{\begin{array}{c} 0\;\;\;\;\;\;\;\;(y_{i}^{n}\leq 0)\\ y_{i}^{n}\;\;\;\;\;\;\left(y_{i}^{n}>0\right), \end{array}\right.$$

where $y_{i}^{n}$ represents the internal value of the ith neuron at the nth layer. The ith neuron of the output layer (the Nth layer) is activated by the softmax function described as follows:

(5) $$z_{i}^{N}\left(y_{1}^{N},\ldots ,\;y_{K}^{N}\right)=\frac{e^{y_{i}^{N}}}{\sum_{k=1}^{K}e^{y_{k}^{N}}},\;\;\;\;\;\;\;\;(i=1,\;2,\;\ldots ,\;K)$$

where K is the number of neurons at the output layer, i.e., the number of categories to be classified.

A CNN is trained by updating the values of weights between neighboring layers based on an error backpropagation (BP) algorithm. The error E, which is called the loss function, for a dataset is defined as follows:

(6) $$E=-\sum_{i=1}^{K}T_{i}\log z_{i}^{N},$$

where $t_{i}$ corresponds to the true value that the CNN should output. To minimize the E value, a stochastic gradient descent (SGD) algorithm [29] is widely used. 

In the present study, we constructed a CNN to classify ripple firings into five categories: “Restraint stress”, “Contact with a female rat”, “Contact with a male rat”, “Contact with a novel object”, and “Before the experiencing of each episode”.

Our preliminary study [22] demonstrated that ripple firings related to the respective episodes “Contact with a female rat” and “Contact with a male rat” were similar in the context of the cross-correlation function. According to the results, we expect that a CNN will find certain features of ripple firings in the five categories and classify them accurately. 

**Figure 3:**
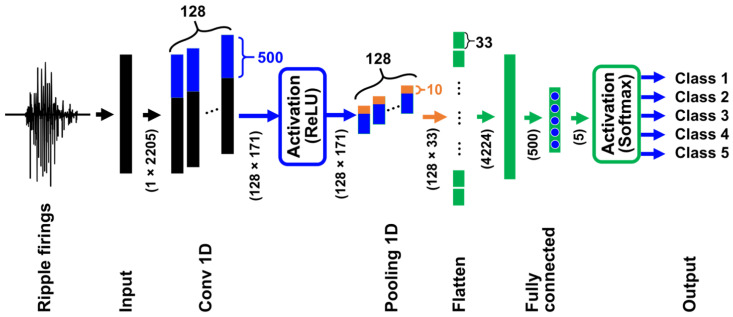
**Structure of the constructed CNN**. The blue and orange rectangles represent the filters in Conv 1D and Pooling 1D layers. The filter sizes are 500 and 10. The numbers in parentheses correspond to the number of nodes at each layer.

We illustrate the architecture of our CNN in Figure 3. The CNN has six layers including input and output layers. The numbers in parentheses represent the number of neurons and their arrangement, i.e., the input layer has 1×2205 neurons. Since the lengths of ripple firings extracted from MUA data are different, we performed zero-padding so that the length of all the ripple firings becomes 2205. The following convolution layer labeled with “Conv 1D” has 128 filters and 171 outputs that are activated by the ReLU in Equation (4), and the max-pooling layer with “Pooling 1D” has 128 filters and 33 outputs. The layer with “Flatten” has the role of converting a 2D layer to a 1D layer and has 4224 neurons; two fully connected layers have 500 and 5 neurons, respectively. The outputs of the last fully connected layer are equal to the number of categories and are activated by the softmax function in Equation (5).

We note that standardized ripple firings without logarithmic transformation ($y_{i}$s) are used for training and testing the CNN. This is because, as mentioned in Section 2.4, our method selects only ripple firings that are not influenced by large spikes.

To minimize the value of the cross-entropy loss function in Equation (6), we trained the constructed CNN by mini-batch training using the SGD algorithm. A mini-batch corresponds to a subset in the dataset for training. In an epoch of mini-batch SGD, all the training data are randomly divided so that the same data are not included in different mini-batches. The CNN is trained for all the mini-batches based on the error BP algorithm, and then the epoch counter is incremented by one. Mini-batches are shuffled every epoch. Training results of CNNs are affected by the size of mini-batches. Generally, it is said that increasing the mini-batch size makes the training of CNNs more robust to outliers. We set the mini-batch size, the number of epochs, the learning rate, and the coefficients of momentum term and weight decay to 64, 400, 0.001, 0.5, and 0.005, respectively.

### 2.6. Gradient-Weighted Class Activation Mapping

Grad-CAM [24] can visualize the basis on which trained CNNs classify input data into each category. Grad-CAM creates heatmaps based on category-specific gradients with respect to feature maps in a certain convolution layer. Analyzing heatmaps related to each ripple-firing waveform can contribute to finding representative partial waveforms in each category.

**Figure 4:**
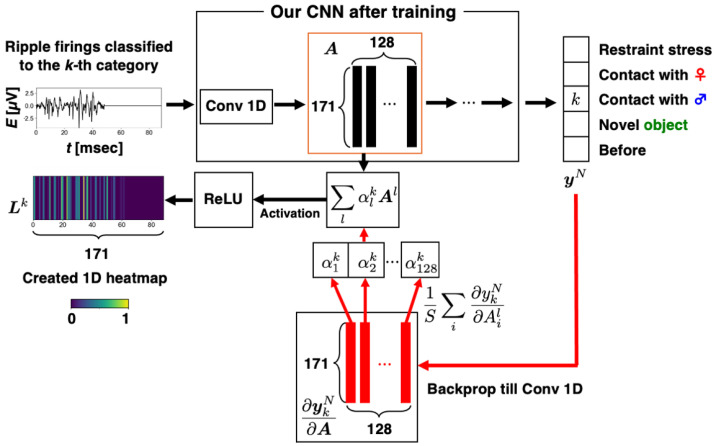
**Schematic diagram of how Grad-CAM creates a heatmap.** A ripple-firing waveform is input to a trained CNN. Feature maps A and an output vector $y^{N}$ are calculated. Based on their gradients, an importance vector $\alpha^{k}$ is computed. After activation by ReLU, a 1D heatmap $L^{k}$ for the input ripple-firing waveform is created.

Figure 4 illustrates a schematic diagram of how Grad-CAM creates a heatmap for a ripple-firing waveform input to our CNN. For an input ripple-firing waveform, we calculate feature maps $A\in R^{171\times 128}$ in the Conv 1D layer of our CNN. The feature maps are associated with the degree of importance $\alpha_{l}^{k}\;(k=1,2,\ldots ,5,\;l=1,2,\ldots ,128)$ for the kth category that the CNN outputs $y_{k}^{N}$, where R expresses the real space and l represents the index of filters in the Conv 1D layer. The importance is defined as follows:

(7) $$\alpha_{l}^{k}=\frac{1}{S}\sum_{i}\frac{\partial y_{k}^{N}\;}{{\partial A}_{i}^{l}},$$

where N represents the last layer number of our CNN and i denotes the index of neurons in the lth filter in the Conv 1D layer, i.e., i=1,2,…,128; S means the size of a feature map whose size is the same as a heatmap to be created, i.e., S=171. Therefore, the value of $\alpha_{l}^{k}$ corresponds to the mean of $\partial y_{k}^{N}/{\partial A}_{i}^{l}$. According to the values of $\alpha_{l}^{k}$, the pixel values in a 1D heatmap for the *k*th category $L^{k}\in R^{171}$ are computed as follows:

(8) $$L^{k}=\mathrm{ReLU}\left(\sum_{l}\alpha_{l}^{k}A^{l}\right),$$

where ReLU is an activation function with the rectified linear unit. Thus, a 1D heatmap for a ripple-firing waveform input to our CNN is created, where the values of $L^{k}$ are normalized by $\max\left(L^{k}\right)$ of every heatmap; we colored all pixels of the heatmap using a colormap “viridis” in matplotlib [30], which is a Python library. To make heatmaps easier to view, we converted 1D heatmaps to 2D-like images by enlarging 1D heatmaps vertically.

From heatmap regions with high pixel values, we can find partial waveforms that our CNN attends to classify the input ripple-firing waveform into correct categories. Investigating heatmaps is helpful for identifying episode-specific waveforms.

## 3. Results

**Figure 5:**
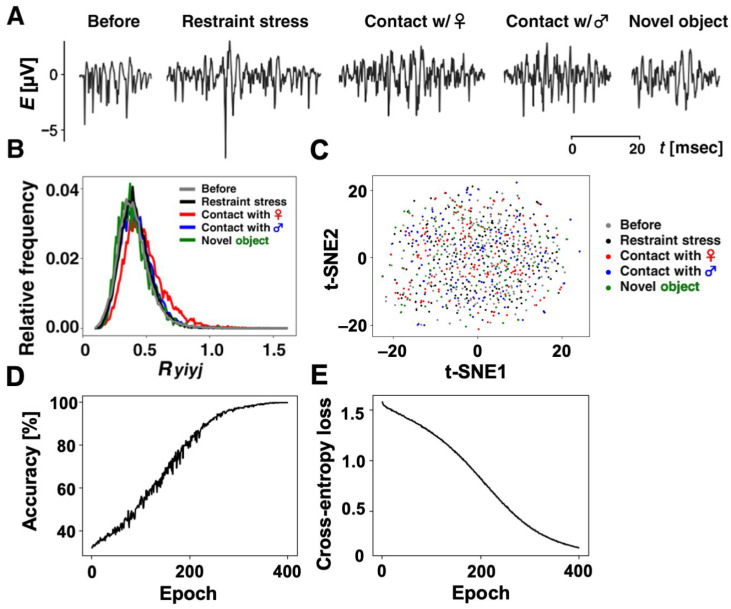
**Classified ripple firings and the learning curves of a CNN.** (**A**) Representative examples of ripple firings classified into 5 categories. (**B**) Frequency distribution of *Ryiyj* values in each category. (**C**) Distribution of ripple firings in a 2D feature space using the t-SNE method. Development of accuracy (**D**) and cross-entropy loss (**E**) for 810 training datasets.

Using our method, we selected 860 similar ripple firings from thousands of ripple firings. Specifically, we have 196 ripple firings in “Restraint stress”, 173 ripple firings in “Contact with a female rat”, 131 ripple firings in “Contact with a male rat”, 93 ripple firings in “Contact with a novel object”, and 267 ripple firings in “Before the experiencing of each episode”. Some of the selected ripple firings in each category are shown in Figure 5A. In addition, we annotated the 860 ripple firings with the five categories so that the annotations {1, 0, 0, 0, 0}, {0, 1, 0, 0, 0}, {0, 0, 1, 0, 0}, {0, 0, 0, 1, 0}, and {0, 0, 0, 0, 1} correspond to the respective categories “Restraint stress”, “Contact with a female rat”, “Contact with a male rat”, “Contact with a novel object”, and “Before the experiencing of each episode”.

Before training our CNN, we described a preliminary analysis for the selected ripple firings. For the ith and jth ripple firings, $y_{i}(t)$ and $y_{j}(t)$, in each category after standardization by Equation (2), we calculated the values of the cross-correlation function as follows:

(9) $$R_{y_{i}y_{j}}=\frac{1}{L}\underset{\tau }{\max}\sum_{t}y_{i}\left(t\right)y_{j}(t-\tau ),\;\;\;\;\;\;\;\;i\neq j$$

where L expresses the length of discrete time that $y_{i}(t)$ and $y_{j}(t-\tau )$ overlap, except for the period of zero-padding. Figure 5B illustrates frequency distributions for $R_{y_{i}y_{j}}$ values in each category. As shown in Table 1, the mean in each category, ${\bar{R}}_{y_{i}y_{j}}$, was calculated as 0.4166, 0.4831, 0.4239, 0.4037, and 0.4037 for “Restraint stress”, “Contact with a female rat”, “Contact with a male rat”, “Contact with a novel object”, and “Before experiencing of each episode”, respectively. The number of samples means the square of the number of ripple firings datasets minus the number of ripple firings datasets. For example, in the category of restraint, $38,220=196^{2}-196$. At first glance, the ${\bar{R}}_{y_{i}y_{j}}$ values for each category were not significantly different among the five groups.

Since a couple of ${\bar{R}}_{y_{i}y_{j}}$ values were similar, we tested them using one-way analysis of variance (ANOVA) and post-hoc Scheffé’s method [31]. The results of our analysis are shown in Table 2. When we set the significance level as 1% or 5%, the critical values were 3.32 or 2.37, respectively. For either case, the F value was 2045.58, which was larger than the critical values, i.e., the one-way ANOVA indicated a significant difference between any two categories. However, as a result of post-hoc tests using Scheffé’s method, no significant difference was observed between ${\bar{R}}_{y_{i}y_{j}}$ values in the “Contact with a novel object” and “Before experiencing of each episode” categories under both the significance level of 1% and that of 5%.

We prepared 860 datasets consisting of ripple-firing waveforms after standardization and annotations in which there were 810 datasets for the training of our CNN and 10 datasets per category for testing. To grasp the characteristic of the 860 datasets before training our CNN, we measured the clustering of datasets within each category and the separation between any two categories. Using t-SNE, which is a method to reduce the dimension of signals, we mapped the 860 ripple firings with the length of 2205 onto a two-dimensional feature space. Figure 5C shows the distribution of points in the feature plane in which each point corresponds to each ripple-firing waveform and the axes express certain features extracted by t-SNE method. In Figure 5C, the average Euclidean distances between each dataset and the centroid within each category “Restraint stress”, “Contact with a female rat”, “Contact with a male rat”, “Contact with a novel object”, or “Before the experiencing of each episode” were computed as 12.53, 11.61, 11.30, 12.96, or 11.36, respectively. We also computed the Euclidean distances between centroids of any two categories in Table 3. Comparing with the average variations within each category, all the distances between categories were quite small. The results imply the difficulty of classification for the datasets.

We first trained our CNN for the 810 datasets. Figure 5D,E show the learning curve of the CNN. The accuracy rate gradually increased as the epoch progressed and became 99.63% at the 400th epoch. Moreover, the value of cross-entropy loss slightly oscillated due to randomly selected mini-batches for every epoch but gradually decreased and became 0.13 at that epoch. The learning curves demonstrated that our CNN was trained so that almost all the training datasets can be classified into correct categories.

After the training, the CNN achieved the accuracy rate of 72.00% for the 50 test datasets. To evaluate the performance of our CNN in detail, we created a confusion matrix for the test datasets. According to the matrix in Table 4, the values of precision, recall, balance accuracy, F1-score, and area under the curve (AUC) were calculated as 0.72, 0.72, 0.72, 0.72, and 0.90, respectively. All the values except for the AUC value were the same as the accuracy rate for the test datasets, and, besides, the value of AUC was close to 1.0. From the results, we can say that the classification of our CNN for the test datasets was relatively accurate without bias.

The result indicates that our CNN caught certain features related to four experienced episodes and before the experience, i.e., we can extract certain waveforms related to each episode from the current stocks of ripple firings as shown in Figure 5A. To support this, for the 36 test datasets that the CNN correctly classified, we applied Grad-CAM to our CNN and extracted partial waveforms that our CNN focused on for classification.

**Figure 6:**
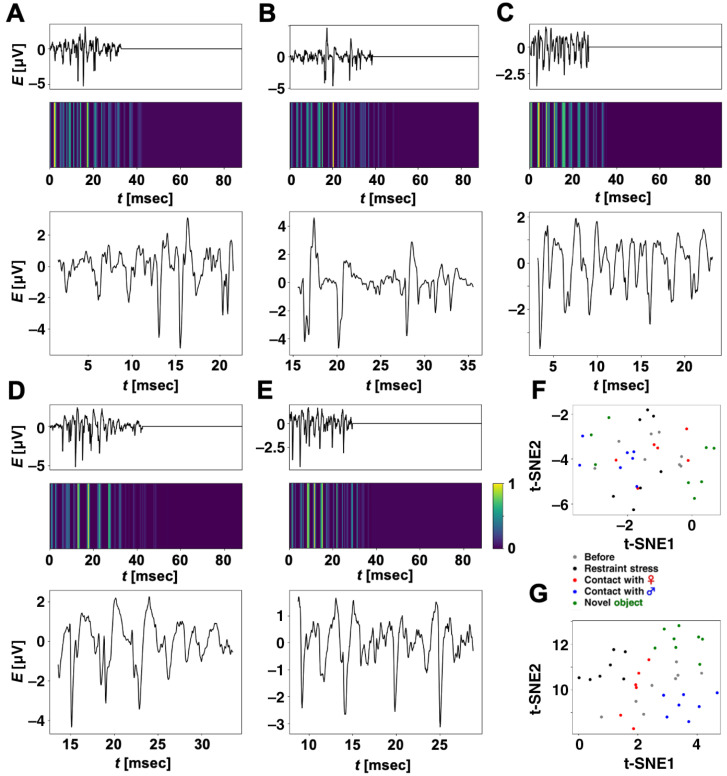
**Analyzed results using Grad-CAM and the t-SNE method.** (**A**–**E**) Ripple-firing waveforms corresponding to test datasets that our CNN correctly classified, heatmaps created by Grad-CAM, and extracted partial waveforms. (**A**) Category with “Restraint stress”. (**B**) Category with “Contact with a female rat”. (**C**) Category with “Contact with a male rat”. (**D**) Category with “Contact with a novel object”. (**E**) Category with “Before the experiencing of each episode”. (**F**) Distribution of 36 points in 2D feature space using t-SNE. Each point corresponds to input ripple-firing waveforms (test datasets) that our CNN correctly classified. (**G**) Distribution of 36 points in 2D feature space using t-SNE. Each point corresponds to partial ripple-firing waveforms extracted by Grad-CAM.

Figure 6A–E show ripple-firing waveforms (test data) input to our CNN, which the trained CNN classifies into each correct category, and their heatmaps created by Grad-CAM. The input waveforms were zero-padded so that their lengths become 2205. Within the time regions corresponding to zero-padding, all the heatmaps took low values, i.e., our CNN did not focus on the zero-padding regions to classify the input waveforms. We extracted partial ripple-firing waveforms, which our CNN mostly focused on for classification; this means a partial waveform including a time region such that $L_{i}^{k}=1.0$. The time range of the extraction was determined to be 20 ms (500 samples), which corresponds to the filter size in Conv 1D of our CNN. From the results, our CNN focused not only on large spikes but also waveforms with relatively low amplitude and classified the input waveforms into each correct category.

Figure 6F shows the distribution of 36 points converted with the t-SNE method. The points correspond to ripple-firing waveforms (test datasets) that our CNN correctly classified. We calculated five centroids in each category and computed average Euclidean distances between each point, and the centroid in every category “Restraint stress”, “Contact with a female rat”, “Contact with a male rat”, “Contact with a novel object”, or “Before the experiencing of each episode” were computed as 0.48, 0.92, 0.88, 2.11, or 1.05, respectively. Euclidean distances between centroids were also computed as shown in Table 5. Comparing the five average distances with the distances in Table 5, we cannot say that differences among the average distances within each category and the distances between categories were significant. Nevertheless, our CNN correctly classified the 36 test datasets.

In the same way, we also analyzed 36 partial waveforms extracted by Grad-CAM. Figure 6G shows the distribution of the 36 points in a t-SNE feature plane. Five centroids and average Euclidean distances between each point and the centroid within “Restraint stress”, “Contact with a female rat”, “Contact with a male rat”, “Contact with a novel object”, or “Before the experiencing of each episode” were calculated as 0.67, 0.27, 0.67, 0.69, or 1.18, respectively. We also computed distances between centroids as shown in Table 6. Each distance shown in Table 6 was larger than the distance between the same categories shown in Table 5. This indicates that focusing on partial waveforms extracted by Grad-CAM increased the distance between the two categories. Moreover, it can be noticed that all the distances shown in Table 6 were greater than the average distances within each category. These facts suggest that partial waveforms corresponding to the nearest points to each centroid may be representative of each category.

## 4. Discussion

Hippocampal ripple oscillations (140–250 Hz) manifest as distinctive electroencephalogram patterns that play a crucial role in memory consolidation and action planning [15,16]. In addition, high-frequency synchronous firings have been observed alongside these ripple oscillations [17]. In this study, we used a 300 Hz–10 kHz recording band to capture individual firings. Given the predominant synchronization of clustered firings with ripples, we termed these events “ripple firings” for analysis.

The episodic experience was accompanied by the occurrence of spontaneous multiple unit firings (super bursts), followed by an increase in both ripple firings and subsequent silent periods [14]. The ripple events coincided with sharp waves, collectively forming sharp wave-ripple complexes (SPW-Rs). CA1 neurons are known to receive large amounts of neural input from CA3 and their optogenetic suppression blocks SPW-Rs in CA1, inhibiting learning and memory [32]. On the other hand, learning itself prolongs the duration of SPW-Rs [19]. During SPW-Rs, a firing sequence replays the spatial learning experience [20], and spikes within this duration increase with learning [19]. We have previously shown that preceding experiences influence the individual characteristics of ripple firings in an episode-dependent manner, and homology analysis of 66,000 pairs revealed that individual ripple firings are not identical and exhibit episode specificity [33]. These findings suggest that ripple events carry learning-related information and that the firings concurrent with ripples encode the details of preceding experiences.

Similar ripples were selected after standardization and logarithmic transformation for waveforms. The selected ripple-firing waveforms only with standardization without logarithmic transformation were fed to our CNN for training and testing. The time-domain preprocessing helped our CNN to classify ripples accurately. Similarly, frequency-domain preprocessing was also useful for assisting in the classification of waveforms in CNNs. However, since the aim of this study was to find partial ripple-firing waveforms using a CNN and Grad-CAM, time-domain preprocessing was preferable to frequency-domain preprocessing from the viewpoint of simplifying our analyses.

While conventional statistical analyses were unable to extract features to classify 860 selected ripple-firing waveforms into five categories, we successfully employed a CNN to categorize ripple firing based on prior experience, achieving high accuracy. To enhance the role of CNNs in this study and the significance of the results, it is imperative to construct a CNN with a higher generalization ability and evaluate its classification accuracy through cross-validation. Once such a CNN with high performance is constructed, the content of the animal’s most recent episodic experience can be read from the ripple firings of hippocampal CA1 neurons.

Here, we applied a general CNN to classify ripple firings and found that even a simple CNN with only one convolutional layer can accurately classify training and test datasets. The near-correct classification of individual ripple firings into five categories marks a significant advance in neuroscience and provides evidence that recent episodic experiences are represented in hippocampal CA1 neurons. If the features captured by our CNN correspond to local waveforms of ripples, then an explainable artificial intelligence (XAI) technique would be effective in identifying local waveforms and extracting unique firing patterns. To explain classification results for each test dataset, Grad-CAM creates heatmaps based on category-specific gradients with respect to feature maps in a convolution layer that the user selects in which the size of the heatmaps is the same size as the feature map. Although the difficulty in analyzing heatmaps is also high for a deep CNN with many convolutional layers in general, our CNN with only one convolutional layer is useful to simplify the identification of local waveforms.

A t-SNE method can plot ripple-firing waveforms and their partial waveforms as points in a 2D feature space. Analyzing the distribution of points and the characteristics of each cluster in the feature plane helps us to find representative local waveforms. Therefore, identifying representative local waveforms in each category is expected by analyzing created heatmaps and the distribution of points mapped by t-SNE.

As the first step to finding representative partial waveforms related to the experience or pre-experience of each episode, we intentionally excluded ripples with low similarity in this study. However, we cannot deny that episode-specific local waveforms may be contained even in ripples with low similarity. In the next step of this study, we should investigate all the stock of ripples in detail.

## 5. Conclusions

Based on previous studies [13,14,33], we hypothesized that the waveform of ripple firings generated by hippocampal CA1 neurons changes dynamically before and after experiencing an episode and that the waveform depends on the type of episode experienced. To confirm this hypothesis, we considered classifying current stocks of ripple firings into five categories consisting of experiencing one of four episodes or before experience. 

As a first step, here, we constructed a CNN to classify the ripple firings. After training, the CNN was able to accurately classify the 810 ripples for training with an accuracy of 99.63% and a cross-entropy loss of 0.13. We also evaluated our CNN for 50 test datasets. The test ripples were classified under an accuracy of 72.00%. According to the confusion matrix in Table 4, the values of precision, recall, balance accuracy, F1-score, and AUC were 0.72, 0.72, 0.72, 0.72, and 0.90, respectively. The results show that our CNN was able to classify the test datasets without bias.

Using Grad-CAM, we subsequently extracted partial waveforms from test waveforms of ripple firings that our CNN correctly classified. The t-SNE method mapped extracted partial waveforms onto points in a 2D feature space. For 36 test datasets that our CNN classified into correct categories, each distance between two centroids for partial waveforms extracted by Grad-CAM was larger than that for whole waveforms input to our CNN. The extracted partial waveforms in Figure 6A–E correspond to the nearest point to the centroids of each category in Figure 6G. The results suggest that ripple firings have certain features related to the experience or pre-experience of each episode, and, besides, it is expected that the partial waveforms in Figure 6A–E are representative of each category. We hypothesized that these features correspond to typical local waveforms. As a next step in this study, we will investigate our stocks of ripple firings carefully to find typical local waveforms using CNNs, Grad-CAM, and t-SNE.

## Figures and Tables

**Table 1:** **Basic statistics for groups of cross-correlation between selected ripple firings per category.** The number of samples means the square of the number of ripple firings datasets minus the number of ripple firings datasets.

	Mean	SEM	Number of Samples
Restraint stress	0.4166	0.0006	38,220
Contact with Female	0.4831	0.0009	29,756
Contact with Male	0.4239	0.0010	17,030
Novel object	0.4037	0.0013	8556
Before	0.4037	0.0005	71,022

**Table 2:** **Results of one-way ANOVA and post-hoc Scheffé’s method**.

	Value of Cross-Correlation Function
Overall	$F_{4,\;164579}=2045.6,\;p<0.001$
Restraint vs. Female	p<0.001
Restraint vs. Male	p<0.001
Restraint vs. Object	p<0.001
Restraint vs. Before	p<0.001
Female vs. Male	p<0.001
Female vs. Object	p<0.001
Female vs. Before	p<0.001
Male vs. Object	p<0.001
Male vs. Before	p<0.001
Object vs. Before	p=0.999

**Table 3:** **Distance between categories for 860 datasets in t-SNE space**.

	Restraint	Female	Male	Object	Before
Restraint	—	2.168	0.804	1.763	1.571
Female	—	—	1.490	0.559	2.782
Male	—	—	—	1.004	1.490
Object	—	—	—	—	2.225

**Table 4:** **Confusion matrix for classification of test data**.

	Predicted Categories					
Correct categories		Restraint	Female	Male	Object	Before
	Restraint	7	1	2	0	0
	Female	2	6	0	0	2
	Male	1	1	7	0	1
	Object	0	1	0	8	1
	Before	1	0	1	0	8

**Table 5:** **Distance between categories for 36 test datasets in t-SNE space**.

	Restraint	Female	Male	Object	Before
Restraint	—	0.507	0.784	0.660	0.412
Female	—	—	1.278	0.265	0.195
Male	—	—	—	1.443	1.137
Object	—	—	—	—	0.456

**Table 6:** **Distance between categories for 36 extracted partial waveforms in t-SNE space**.

	Restraint	Female	Male	Object	Before
Restraint	—	1.424	3.119	2.804	1.970
Female	—	—	1.778	2.685	0.773
Male	—	—	—	2.783	1.163
Object	—	—	—	—	2.208

## Data Availability

The entirety of raw data from this study is available from the authors upon request. The data are not publicly available due to the inclusion of data for ongoing research.
